# Spatial Interaction of Archaeal Ammonia-Oxidizers and Nitrite-Oxidizing Bacteria in an Unfertilized Grassland Soil

**DOI:** 10.3389/fmicb.2015.01567

**Published:** 2016-01-22

**Authors:** Barbara Stempfhuber, Tim Richter-Heitmann, Kathleen M. Regan, Angelika Kölbl, Pia K. Wüst, Sven Marhan, Johannes Sikorski, Jörg Overmann, Michael W. Friedrich, Ellen Kandeler, Michael Schloter

**Affiliations:** ^1^Environmental Genomics, Helmholtz Zentrum München, German Research Centre for Environmental HealthNeuherberg, Germany; ^2^Faculty of Biology/Chemistry, University of BremenBremen, Germany; ^3^Institute of Soil Science and Land Evaluation, University of HohenheimStuttgart-Hohenheim, Germany; ^4^Lehrstuhl für Bodenkunde, Technische Universität MünchenFreising, Germany; ^5^Leibniz-Institute DSMZ, German Collection of Microorganisms and Cell CulturesBraunschweig, Germany

**Keywords:** nitrification, ammonia oxidation, nitrite oxidation, niche separation, spatial analysis, grassland

## Abstract

Interrelated successive transformation steps of nitrification are performed by distinct microbial groups – the ammonia-oxidizers, comprising ammonia-oxidizing archaea (AOA) and bacteria (AOB), and nitrite-oxidizers such as *Nitrobacter* and *Nitrospira*, which are the dominant genera in the investigated soils. Hence, not only their presence and activity in the investigated habitat is required for nitrification, but also their temporal and spatial interactions. To demonstrate the interdependence of both groups and to address factors promoting putative niche differentiation within each group, temporal and spatial changes in nitrifying organisms were monitored in an unfertilized grassland site over an entire vegetation period at the plot scale of 10 m^2^. Nitrifying organisms were assessed by measuring the abundance of marker genes (*amoA* for AOA and AOB, *nxrA* for *Nitrobacter*, 16S rRNA gene for *Nitrospira*) selected for the respective sub-processes. A positive correlation between numerically dominant AOA and *Nitrospira*, and their co-occurrence at the same spatial scale in August and October, suggests that the nitrification process is predominantly performed by these groups and is restricted to a limited timeframe. Amongst nitrite-oxidizers, niche differentiation was evident in observed seasonally varying patterns of co-occurrence and spatial separation. While their distributions were most likely driven by substrate concentrations, oxygen availability may also have played a role under substrate-limited conditions. Phylogenetic analysis revealed temporal shifts in *Nitrospira* community composition with an increasing relative abundance of OTU03 assigned to sublineage V from August onward, indicating its important role in nitrite oxidation.

## Introduction

Nitrification has been the focus of many studies over decades due to the ecological importance of this process, especially for agricultural ecosystems. Nitrification determines, to a great extent, whether applied fertilizers will function either as plant growth supporting components or as environmental pollutants. Nitrate leaching into water causes eutrophication, and the emission of N_2_O, a highly potent greenhouse gas, contributes to climate change (Ollivier et al., 2011). However, results of the relative contributions of key players have been contradictory – supportive either of archaeal (Leininger et al., 2006; Adair and Schwartz, 2008; Zhang et al., 2012) or bacterial ammonia-oxidizer (Di et al., 2009; Jia and Conrad, 2009) dominance – or have suffered from missing links between abundances of nitrifiers and nitrification activities (Di et al., 2009). These discrepancies can be explained in part by the designs of those studies, which have focused mainly on detailed analyses of key players involved in one or another sub-process, thereby neglecting to account for the fact that nitrification requires a strong interaction among phylogenetically differing microbes with different ecophysiologies.

The first steps, the oxidation of ammonia to hydroxylamine and nitrite, can be catalyzed by ammonia-oxidizers. The last step of the transformation process, the oxidation of nitrite to nitrate, is performed by a distinct group of organisms, the nitrite-oxidizers (Konneke et al., 2005).

Ammonia-oxidizers comprise both ammonia-oxidizing bacteria (AOB) and archaea (AOA) (Kowalchuk and Stephen, 2001; Treusch et al., 2005). Their abundances have been monitored in a wide range of ecosystems (Ochsenreiter et al., 2003; Francis et al., 2005; Treusch et al., 2005; Stahl and de la Torre, 2012). The discovery of archaeal involvement in ammonia-oxidation (AO), the frequent numerical dominance of AOA over AOB, and their active participation in AO (Leininger et al., 2006; De La Torre et al., 2008; Hatzenpichler et al., 2008; Offre et al., 2009; Schauss et al., 2009), have thrust the relative contributions of AOA and AOB into the research spotlight. Several studies have indicated that AOA and AOB colonize different niches in soil (Keil et al., 2011; Ollivier et al., 2013; Regan et al., 2014; Stempfhuber et al., 2014) and differ in their ecophysiologies (Hatzenpichler, 2012); however, their putative interaction partners have remained largely unaddressed (Prosser and Nicol, 2008).

The ability to oxidize nitrite is found in only six bacterial genera: *Nitrobacter, Nitrotoga, Nitrococcus, Nitrospina, Nitrospira*, and *Nitrolancetus*; affiliated to the alpha-, beta-, gamma-, and delta-classes of *Proteobacteria* and the phyla *Nitrospirae* and *Chloroflexi*, respectively (Daims et al., 2001; Bock and Wagner, 2006; Alawi et al., 2009; Attard et al., 2010; Sorokin et al., 2012). Nitrite-oxidizing bacteria (NOB) can be found in a variety of habitats (Abeliovich, 2006), from marine and freshwater aquatic systems (Watson et al., 1986; Stein et al., 2001), to wastewater treatment plants (WWTPs) (Juretschko et al., 1998; Daims et al., 2001; Gieseke et al., 2003; Spieck et al., 2006) and terrestrial ecosystems (Bartosch et al., 2002; Wertz et al., 2012). In terrestrial environments *Nitrobacter* (NB) and *Nitrospira* (NS) have been identified as the dominant genera (Bartosch et al., 2002; Cébron and Garnier, 2005; Kim and Kim, 2006; Ke et al., 2013). Niche differentiation amongst NOB has been proposed in several studies in both aquatic and terrestrial habitats (Schramm et al., 1999; Cébron and Garnier, 2005; Ke et al., 2013; Ollivier et al., 2013; Placella and Firestone, 2013). Shifts between NB and NS have been shown to be a consequence of different strategies related to substrate affinity (Attard et al., 2010). It has been suggested that NB are *r*-strategists, favored under high substrate concentrations owing to lower substrate affinity of their respective catalyzing enzyme. NS, however, as *K*-strategists, are capable of tolerating lower nitrite and oxygen concentrations (Schramm et al., 1999; Daims et al., 2001; Kim and Kim, 2006).

It is commonly assumed that the two transformation steps for complete nitrification are dependent on the interaction of two distinct microbial guilds in terrestrial ecosystems (Kowalchuk and Stephen, 2001). As autotrophic ammonia-oxidizers gain their energy from the conversion of ammonia to nitrite, AOB and NOB are thought to be dependent on each other in a mutualistic relationship. Nitrite, the product of ammonia-oxidation (AO) is available for nitrite-oxidizers as substrate, which, under aerobic conditions, in turn assures the consumption and the removal of the toxic nitrite in the environment by nitrite oxidation (Juretschko et al., 1998; Maixner et al., 2006). Thus, the processes of ammonia- and nitrite-oxidation are considered to be spatially dependent (Grundmann et al., 2001). Studies on the interactions and spatial structure of AOB and NOB have been performed mainly in aquatic systems or biofilm- and activated sludge-based WWTPs (Gieseke et al., 2003; Ke et al., 2013). In soils, the number of studies on interactions between ammonia- and nitrite-oxidizers is limited, suggesting an interaction of AOB with both NS- and NB-like NOB, and co-occurrence of AOA with NS (Xia et al., 2011; Wertz et al., 2012; Ke et al., 2013; Ollivier et al., 2013; Daebeler et al., 2014). Studies which take spatial and temporal dynamics of these nitrification networks into account, are, however, missing.

Hence, the focus of this study was to investigate the formation of networks of ammonia- and nitrite-oxidizers as influenced by season in a grassland soil. We postulated that the dominant forms of nitrifying networks are AOB – NB under high substrate concentrations in spring and summer and AOA – NS under lower substrate concentrations in autumn. As AOA (Jia and Conrad, 2009; Tourna et al., 2011; Daebeler et al., 2014) and NS (Daims et al., 2001; Lücker et al., 2010; Lebedeva et al., 2013) are considered to be mixotrophs, both groups may act also independently, mainly at locations with high carbon availability. To test our hypotheses, we followed the seasonal dynamics and spatial distribution patterns of AOA, AOB, NB, and NS using qPCR-based approaches to assess the abundance of marker genes for each group. We then linked these data to ammonia and nitrate availability. The dynamics of metabolically active NOB were further analyzed by screening the 16S rRNA inventory (obtained by barcoded Ilumina sequencing) both to gain a deeper insight into the active community structure of NOB as affected by time and space, and to link these to the presence of AOA and AOB.

## Materials and Methods

### Study Site Description and Sampling Design

The experiment was performed in the frame of the ‘German Biodiversity Exploratories’^1^ (Fischer et al., 2010), a large interdisciplinary study aimed at improving our understanding of the effects of land use intensity on diversity at different scales. A low land-use intensity grassland site (48°25′0.01″ N, 9°30′0.00″ E), which did not receive additional fertilizer input and was subjected only to short-term grazing in the Biosphere Reserve Schwäbische Alb in the South-west of Germany, was selected for this study (Regan et al., 2014). Mean annual temperature in the year of sampling was 8.1°C; mean annual precipitation was 810 mm. The experimental site (plot ID: AEG31) was classified as Rendzic Leptosol (according to the FAO classification system). Abiotic soil parameters such as pH, carbon and nitrogen content, bulk density and soil texture were stable during the season.

In an unfertilized grassland site, a 10 m × 10 m plot was divided into 30 subplots (each 2 m × 1.67 m). Six pairs of sampling locations were randomly assigned within each subplot, each pair separated by 50 cm to provide appropriate lag distances for later geostatistical analyses. One pair from each subplot was sampled at each of six dates over one growing season. In total, 360 samples were collected in April, May, June, August, October, and November 2011 (60 per date × 6 dates). Dates were chosen to correspond to stages of plant growth on the plot. Per date, two samples were collected from the upper 10 cm soil horizon from each of the 30 subplots within the 10 m × 10 m plot (i.e., 60 samples per date in total). Soil samples were collected with a soil auger (58 mm diameter) to 10 cm depth. Soil was sieved (5 mm) and homogenized in the field. Samples for DNA extraction were frozen in liquid nitrogen in the field, and stored at -20°C. Detailed information on soil properties and sampling details can be found in the supplemental material or obtained from Regan et al. (2014).

### Extraction of Nucleic Acids

A total of 360 samples were collected at six sampling dates, 60 samples per date, over one growing season, from April to November 2011. All samples were extracted in duplicate from homogenized soil subsamples (0.3 g) using the FastDNA^®^ SPIN Kit for Soil (MP Biomedicals, Solon, OH, USA). Concentrations of the extracts from both sample replicates were measured independently on a NanoDrop^®^ ND-1000 spectrophotometer (Thermo Scientific, Wilmington, DE, USA), then pooled and re-measured to confirm the final DNA concentration. For qPCR measurements, samples were diluted to a target concentration of 5 ng DNA μl^-1^ with ultra-pure water. This concentration has been determined as not inhibiting PCR in pre-experiments (data not shown). Extractions of rRNA from homogenized soil samples were conducted following a protocol modified after Lueders et al. (2004), in which the centrifugation step after addition of PEG was extended to 90 min. The nucleic acids were resuspended in 30 μl EB buffer, and the precipitation of the RNA after DNA digestion was carried out with isopropanol in the presence of sodium acetate.

### Quantification of Marker Genes

Real-time quantitative PCR was performed on a 7300 Real-Time PCR System (Applied Biosystems, Germany) using SyBr Green as fluorescent dye. To quantify abundances of AOA and AOB the respective *amoA* genes were used as target. NS-like and NB-like NOBs were targeted by primer sets for 16S rRNA genes for NS and *nxrA* genes specific for NB. As primers for NS-like *nxrA* genes have been tested and shown to be non-specific (Ke et al., 2013), we chose specific 16S rRNA gene primers to target NS-like NOB. PCRs were performed according to Ollivier et al. (2013), major PCR parameters are listed in Supplementary Table S1. Serial dilutions of the plasmids containing fragments of the marker genes (Supplementary Table S1) were used for standard curve calculations. To determine the specificity and correct fragment size of the amplified qPCR products, a melting curve analysis was conducted after qPCR for each sample, followed by gel electrophoresis on a 2% agarose gel for randomly selected samples. Efficiencies obtained were above 80% and *R*^2^ was determined to be above 0.99 for each qPCR assay.

### Sequencing of 16S rRNA and Phylogenetic Analysis

We used universal primers targeting the 16S rRNA gene, and conducted paired end Illumina sequencing on a HiSeq 2500 (Illumina, San Diego, CA, USA). Besides the specific binding site 341f (Muyzer et al., 1993) and 515R (Lane, 1991), the primers contained the Illumina adapter sequence as well as the binding site for sequencing primers. Additionally, the reverse primer included a barcode region of six nucleotides. Briefly, RNA extracts from soils were reversely transcribed with GoScript (Promega, Madison, WI, USA), and PCR amplification was carried out targeting the V3 region, using primers containing Illumina adapters and a barcode (reverse primer only) (Bartram et al., 2011). Amplicons were purified from agarose gels and cleaned with NucleoSpin Extract II columns (Macherey & Nagel, Düren, Germany) prior to sequencing at the Helmholtz Center for Infectious Diseases, Braunschweig, Germany. Two samples (one in April, one in June) were lost during the process. Sequence raw data were analyzed using a bioinformatic pipeline: downstream processing included the trimming to 100 base pairs for each direction, the removal of contaminating primer dimers, and the joining of the remaining reads. Joined reads were checked for chimeric sequences with UCHIME (Edgar et al., 2011), and then clustered with CD-HIT-OTU for Illumina (Li and Godzik, 2006; Fu et al., 2012). Obtained representative sequences were finally annotated with the RDP-Classifier (Wang et al., 2007), with a similarity threshold of 97% for OTU clustering and a confidence cutoff of 0.5. After the removal of single- and doubletons, the final dataset was created.

For the identification of NOB in the dataset, suitable genera covered by the respective qPCR primer pairs for NS and NB were identified with the Genomatix software suite using the FastM and ModelInspector tool (Klingenhoff et al., 1999). OTUs affiliated exclusively with those genera were then extracted from the 16S rRNA dataset. For reference sequences, the RDP-Classifier (with 16S rRNA training set 10), BLAST (vs. the Nucleotide collection (nr/nt)) (Altschul et al., 1990), and ARB (with the SILVA 119 SSU REF NR database) (Ludwig et al., 2004; Quast et al., 2013) were used to extract type strain sequences and close relatives for phylogenetic analysis. *Nitrospina gracilis*, a marine NOB, was chosen as an outgroup (Luecker et al., 2013). The obtained set of sequences was aligned with JalView (Waterhouse et al., 2009) and the implemented MAFFT algorithm (preset G-INS-i, for maximum accuracy) (Katoh et al., 2005). We first checked the alignment for the best fitting evolutionary model with MEGA 6 (Tamura et al., 2013). The model with the least Bayesian Information Criterion was considered to best describe the substitution pattern, and was subsequently used for tree construction, in this case the Kimura-2 parameter model with gamma distribution (K2+G). Tree topologies were then calculated with the Maximum Likelihood and Neighbor Joining algorithms as implemented in MEGA 6.

The sequence reads analyzed for this manuscript have been uploaded to the Short Read Archive under the project ID “PRJEB10957.” The full study can be accessed under the following link: http://www.ebi.ac.uk/ena/data/view/PRJEB10957.

### Statistics

Statistical analyses were performed using the R environment^2^. To prepare data for statistical analyses, qPCR abundance data were log (x+1) transformed. We conducted pairwise Pearson and Spearman rank correlation analyses between all variables and observations for initial data screening. Selected highly correlated pairs were corrected for autocorrelation by using functions available in the nlme package. First we formulated a null model between two variables with function lme(), then updated this model by using one of five correction procedures for spatial autocorrelation (exponential, spherical, linear, Gaussian, rational quadratic). The best fitting corrections according to the Akaike Information Criterion (AIC) were chosen for the final regression model. For pairwise comparisons of group means between the six sampling dates, we used the function glht() of the package multcomp with method “Tukey” on generalized linear models with the appropriate distribution families for each group of variables (Hothorn et al., 2008; Herberich et al., 2010). Non-random spatial dependence, i.e., the relation of data points in dependency of their distance, was analyzed using the geostatistical approach published by Steffens et al. (2009). A semi-variogram describes the degree of variability as a function of spatial separation of samples (Grundmann and Debouzie, 2000). Spherical models were fitted to each experimental semivariogram using the gstat fitting routine of R. Furthermore, exponential models were tested if no spherical model could be fitted. For underlying equations, see e.g., Steffens et al. (2009). In case no model could be fitted, either the parameter under investigation was homogeneously distributed or the spatial distribution was independent of the scale chosen (see Supplementary Table S3) and thus could not be visualized by kriged maps. More detailed information on our geostatistical approach is provided in the supplemental material. The variogram model was used in order to interpolate the measured data to non-sampled sites within the investigated plot (Steffens et al., 2011) and kriged maps were constructed to visualize the spatial structure of gene abundances at the plot scale. Maps were constructed by ordinary kriging taking advantage of the ArcGIS Software (ArcMap 10.0, ESRI^®^ 2010, Germany) wherever a model could be fitted to the dataset.

## Results

### Temporal Dynamics of Ammonia- and Nitrite-Oxidizers

**FIGURE 1:**
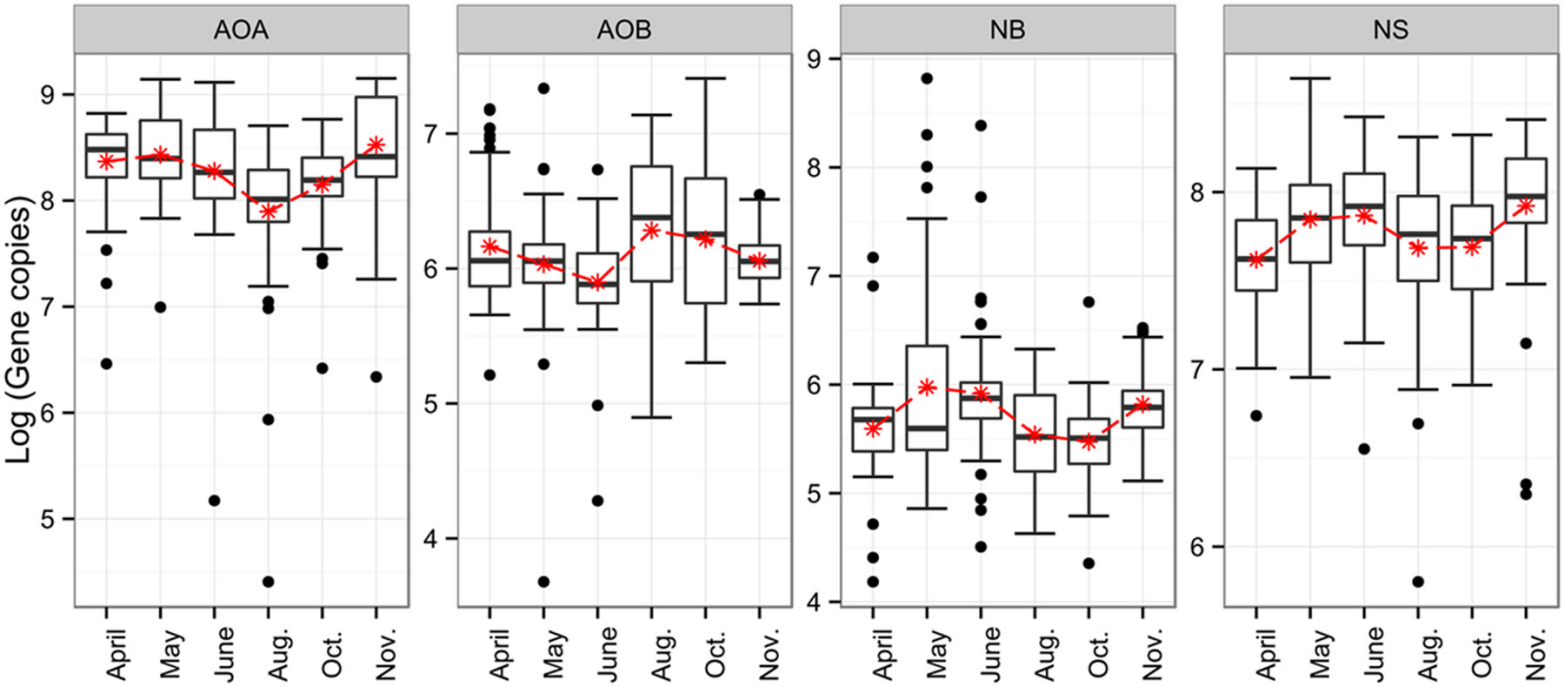
**Boxplots for seasonal dynamics of ammonia- and nitrite-oxidizers.** Depicted are gene copy numbers. AOA, ammonia-oxidizing archaea; AOB, ammonia-oxidizing bacteria; NB, *Nitrobacter*-like; NS, *Nitrospira*-like.

To assess putative temporal changes in the abundances of ammonia- and nitrite-oxidizers, we determined the gene copy numbers of the 16S rRNA gene (NS), *nxrA* (NB) and *amoA* (AOA and AOB) (Supplementary Table S2; **Figure 1**). Numbers of 16S rRNA genes for NS were in the range of 10^7^ to 10^8^ gene copies per g soil dry weight, whereas NB were lower in abundance with 10^5^ to 10^6^ *nxrA* gene copy numbers. Exceptions were a few sampling sites with very high gene copy numbers exceeding 10^7^. Gene copy numbers indicative for NS increased from April to May, and declined slightly in June and August/October when lowest values were detected. In November the abundance of NS-like NOB increased to its maximum. Interestingly, the seasonal dynamics of AOA abundance closely resembled the trend of the NS gene abundance pattern with a decline in August and October and highest values in May and November. AOB abundance, in contrast, exhibited highest gene copy numbers in August and October, coinciding with the lowest gene abundances for AOA and NS; lowest gene copy numbers were detected in May/June and November. Throughout the entire season, AOB copy numbers (in the range of 10^6^) were generally lower than AOA (in the range of 10^8^). In terms of statistical significance, changes in abundance for NS were not significant after the tested model was corrected for spatial autocorrelation. For AOA, AOB, and NB, however, significant changes were found for the June–August transition (*p* < 0.01), as well as for the decrease in AOA (*p* < 0.001) and NB (*p* < 0.05) between October and November, and for NB in early spring (*p* > 0.01).

### Spatial Analysis of Gene Abundances of Ammonia- and Nitrite-Oxidizers

In order to detect spatial structures of the investigated groups at the plot scale of 10 m^2^, geostatistical semivariogram analyses were conducted. Supplementary Table S3 shows semivariogram parameters of gene abundance data for the respective sampling dates. Spherical models could be fitted for all sampling dates for NS-like NOB, whereas spatial dependence was found at only few dates for the other genes.

Range, nugget and sill were determined to assess the spatial behavior of variables (Supplementary Table S3). For most gene abundance data, spatial dependence was captured within the sampling area with seasonally varying ranges of autocorrelations (4.9–12.8 m for AOA, 2.3–9.1 m for AOB, 1.2–21.2 m for NS, 4.5–12.3 m for NB). For some parameters, a far-reaching spatial autocorrelation would be expected when the determined range exceeds the boundaries of the plot as, e.g., for NS-like NOB with a range of 21 m in October, which did not represent a reliable range, because it exceeded the maximum distance between sampling points. Gene abundances of NB in November and NS in April and October exhibited an extremely high spatial dependency (above 87%). For NB, the degree of spatial dependence increased during the year. However, the seasonal dynamics of NS-like NOB first revealed a decline in spatial dependence visible until June, followed by an increase in August and again in November. In October, the highest spatial dependency of about 93% was reached for NS-like NOB. The degree of spatial dependence was rather low for AOA and AOB (between 2.4 and 36.5%) and the data sometimes exhibited a large nugget effect, implying high non-measured small-scale variability.

**FIGURE 2:**
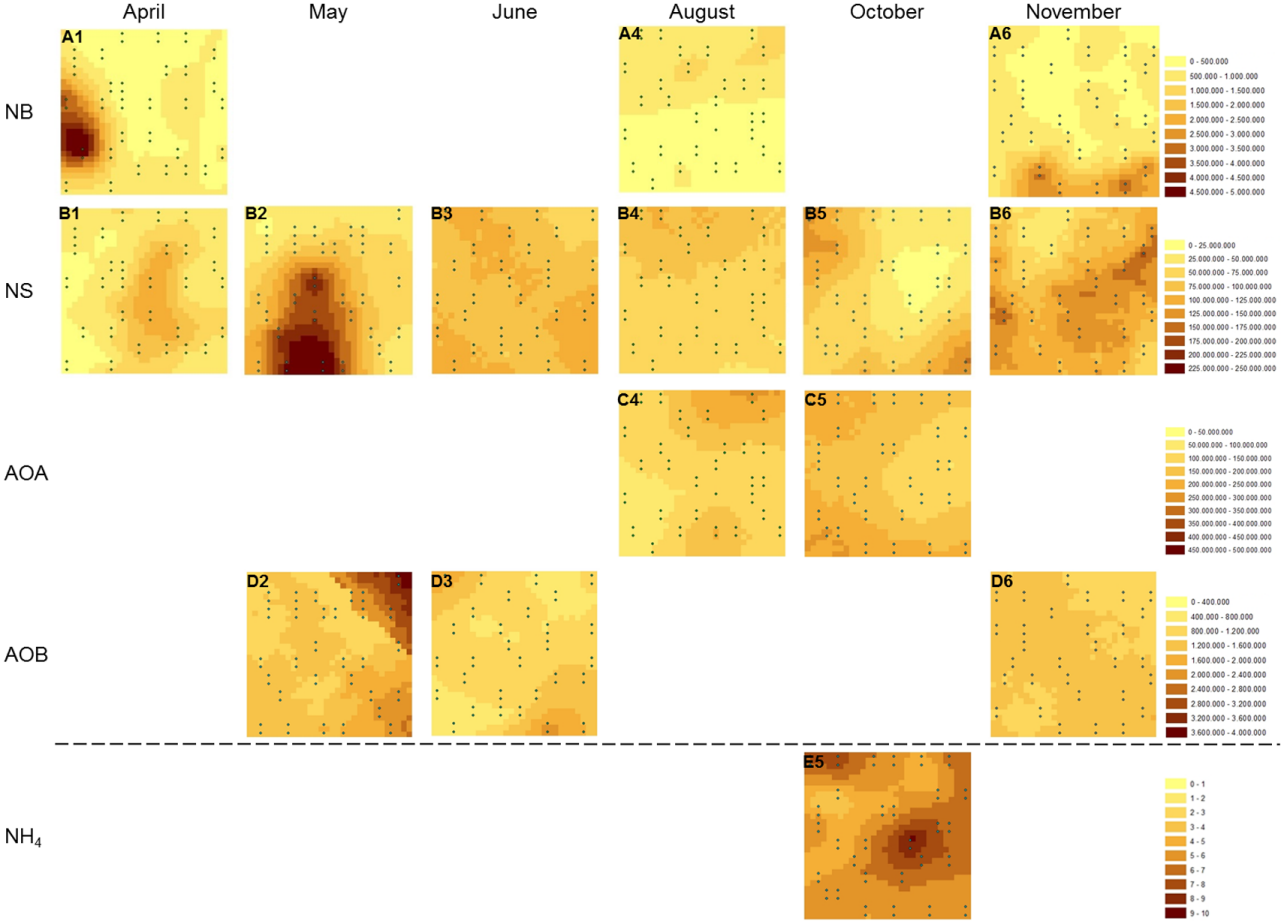
**Spatial distribution of selected variables.** Kriged maps were constructed for gene abundances of **(A)** *nxrA* gene (NB), **(B)** 16S rRNA genes (NS), **(C)** *amoA* gene (AOA), **(D)** *amoA* gene (AOB), and for soil ammonium content **(E)** at different sampling dates (1–6). Gene abundances are given in gene copy numbers per g soil (dry weight), ammonium concentration is given in μg N per g soil (dry weight). AOA, ammonia-oxidizing archaea; AOB, ammonia-oxidizing bacteria; NB, *Nitrobacter*-like nitrite-oxidizing bacteria; NS, *Nitrospira*-like nitrite-oxidizing bacteria.

Kriged maps, used to visualize the spatial distribution of the investigated variables, revealed highly variable spatial distributions over the sampling period for both NB and NS-like NOB (**Figure 2**). In case no map could be constructed, the spatial distribution of the parameter of interest was too homogeneously distributed to be visualized by a spherical model or could not be resolved at our sampling scale. On the sampling dates for which kriged maps could be generated for NB, varying distribution patterns were detected, ranging from medium-sized patches in November (**Figure 2A6**), to large patches with hotspots in April (**Figure 2A1**), and finally more homogeneous structures in August (**Figure 2A4**) with higher abundances in the upper part of the plot interspersed by a few smaller nested patches. Spatial autocorrelation patterns of NS, observed at each sampling date, varied extensively with the season (**Figures 2B1–6**). NS abundance was spatially structured in larger patches with rather smooth transitions from areas of low to high abundance in April and May, the latter even harboring a pronounced hot spot of high abundance. This rather homogeneous distribution changed to more small-scale patchiness with a heterogeneous structure in June. In August, a continuous decline in abundances located at the upper border of the plot was evident, again becoming more homogeneous, with larger patches in October and lowest values in the right half of the plot. Pronounced small-scale heterogeneity with a relatively high number of small sharply zoned patches could be demonstrated for NS-like NOB in November; AOA distributions could be displayed in August and October (**Figures 2C4,5**) revealing larger homogeneous patchiness with gradient-like structures of gene abundances. AOB gene abundance was more heterogeneously distributed in May than in the other months with smaller patches and a more pronounced gradient-like structure in the upper right corner of the plot (**Figures 2D2,3**). Spatial variability was more homogeneous in November. **Figure 2E5** shows the spatial distribution of NH_4_^+^ with a pronounced large patch of high concentration on the right side of the plot, corresponding to the lowest abundances for AOA and NS gene copy numbers measured at this sampling date.

### Phylogenetic Analysis of Active Nitrite-Oxidizing Bacterial Community Composition

To further differentiate the various groups of active NOB, a 16S rRNA based barcoding approach was performed and OTUs affiliated with selected NOB groups (NS and NB) were further analyzed. In the 16S rRNA dataset, we detected 40 OTUs assigned to genus *Nitrobacter* based on 97% sequence similarity of the variable region 3, but a single OTU accounted for more than 99% of reads associated with this genus. This particular OTU also was the second most abundant signal in the entire dataset and was represented by 5.4 million reads (∼1.1% of the entire bacterial dataset). For the phylum *Nitrospira*, 285,000 reads (0.063% of all bacterial reads) could be assigned to 36 OTUs. However, 33 of these OTUs were found to be spurious, hence, we focused on the remaining three generalist OTUs in this phylum, which accounted 99.3% of all NS assigned reads and appeared in all samples. The three representative sequences for these OTUs exhibited sequence similarities between 92% (01 vs. 03), 93% (02 vs. 03), and 97% (01 vs. 02), respectively.

The relative abundance of the NB OTU strongly increased from April to May (*p* < 0.001) and from August to October (*p* < 0.01), when this OTU reached its annual maximum, decreasing significantly again between October and November (*p* < 0.05), maintaining relatively constant levels between May and August (Supplementary Figure S1). This NB-OTU at some dates exhibited very high correlation to the NS-OTUs (especially in April and August). Relative abundances of the three NS-OTUs were stable during the first three sampling dates of the year. For all three OTUs, the abundances increased from June to August (*p* < 0.05), except OTU01, which was not significant (*p* = 0.06). Interestingly, the activities of OTUs 01 and 02 both declined during the late season sampling dates, whereas OTU03 remained stable, thus increasing its abundance compared to the other *Nitrospira* OTUs (Supplementary Figure S1).

*Nitrospira* OTUs showed overall positive correlations with each other (OTU01-02: *r* = 0.683, OTU01-03: *r* = 0.530, OTU02-03: *r* = 0.512), with varying strengths of correlations if the sampling dates were analyzed separately (Supplementary Figure S2). In accordance with their sequence-based similarity of 97%, OTU01 and 02 were highly correlated at most of the sampling dates (*r* > 0.650). Correlations with NS OTU03 were generally weaker, but still significant. NS OTUs did not show any correlation to ammonium (Supplementary Figure S2). At the beginning and toward the end of the year, significant correlations of NS OTUs with nitrate content were found, especially for OTU 02 (up to *r* = 0.42 in November). A weak correlation between nitrate and the *Nitrobacter*-OTU was also found in October.

**FIGURE 3:**
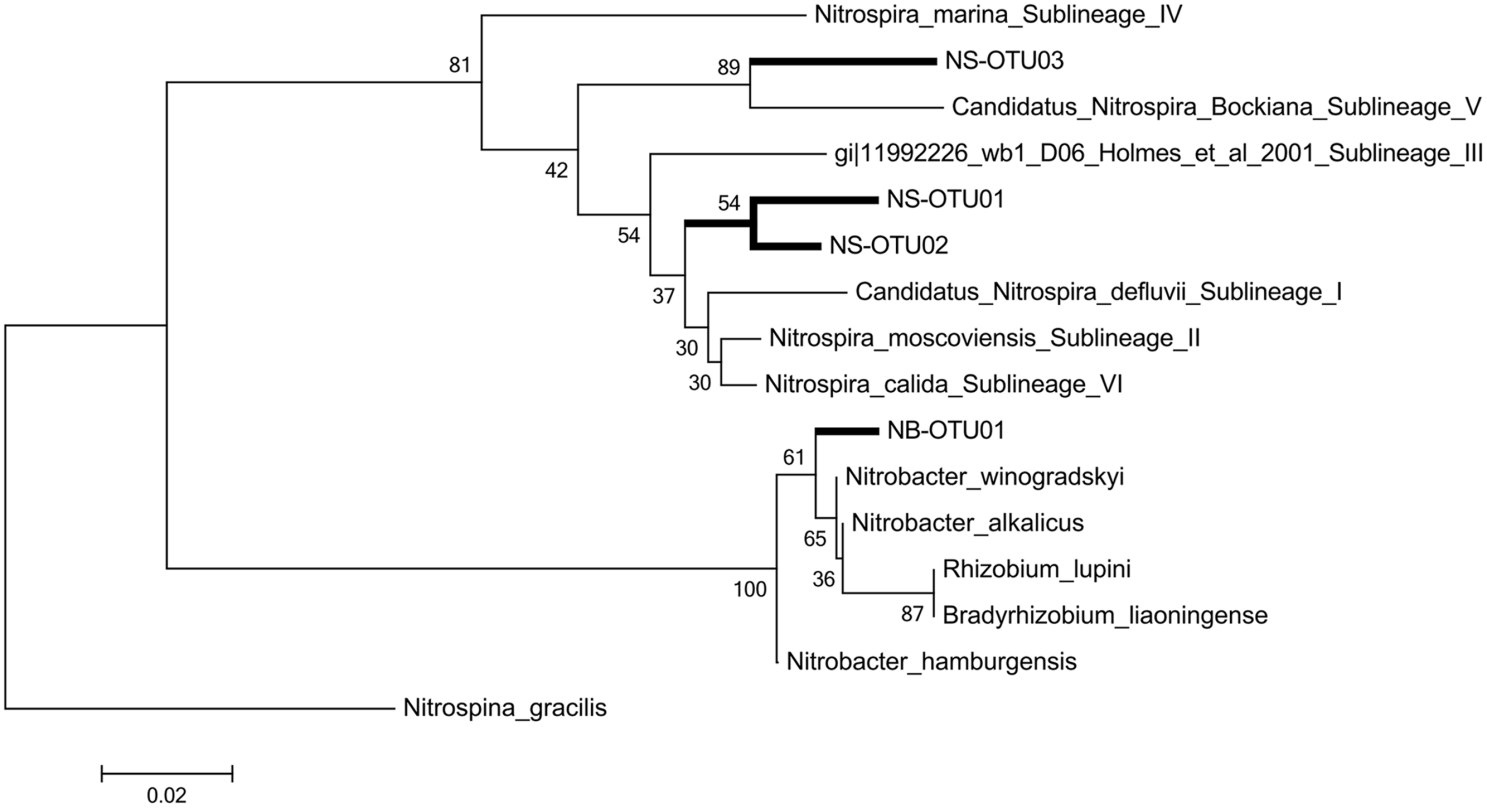
**Phylogenetic tree.** The evolutionary history was inferred using the Neighbor-Joining method. The optimal tree with the sum of branch length = 0.39985022 is shown. The percentage of replicate trees in which the associated taxa clustered together in the bootstrap test (100 replicates) and are shown next to the branches. The evolutionary distances were computed using the Kimura 2-parameter method with gamma distribution (K2+G) and are in the units of the number of base substitutions per site. The analysis involved 10 nucleotide sequences. All positions with less than 10% site coverage were eliminated. That is, fewer than 90% alignment gaps, missing data, and ambiguous bases were allowed at any position. There were a total of 182 positions in the final dataset. Evolutionary analyses were conducted in MEGA6. Sequences contain sublineage designations as given in Daims et al. (2001).

**FIGURE 4:**
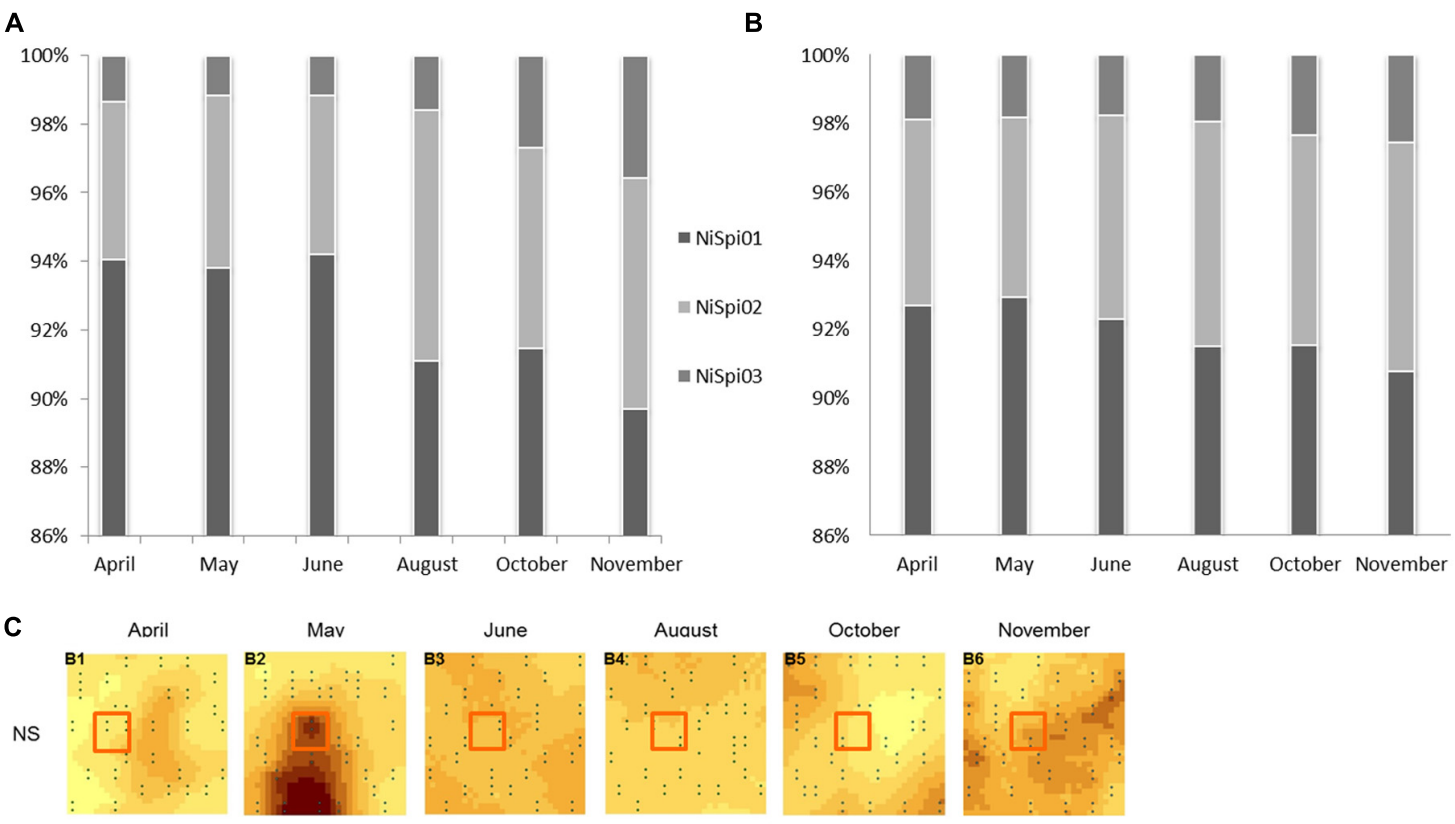
**Relative abundances of NS-assigned OTUs.** Columns display the relative abundances of *Nitrospira*-like NOB OTUs 01-03 over the season. The total abundance of NS-assigned OTUs was set to 100%. NS = *Nitrospira*-like nitrite-oxidizing bacteria. Barcharts depict either relative abundances within one selected subplot **(A)** or represent the complete dataset **(B)**. The location of the selected subplot is indicated by the red square **(C)**.

A phylogenetic tree was constructed based on the Neighbor Joining algorithm (**Figure 3**) and detailed examinations were performed on the affiliation of the NS OTU-sequences to sublineages of NS-like NOB, as designated in Daims et al. (2001) and Lebedeva et al. (2011) (Supplementary Table S4). The topology of the neighbor joining tree was further confirmed by the maximum likelihood method (data not shown). NS OTU01 and OTU02 were located in proximity to sublineages I, II and VI. It is of note that for some taxa, the variable region 3 of the 16S rRNA cannot clearly resolve the sequence affiliation beyond the genus level, which seemed to happen in the case of some of the sublineages. Both conducted methods, however, place NS OTU03 with a similarity level of 94% in the sublineage V of *Nitrospira* with *Ca. Nitrospira bockiana* as cultured representative. To determine whether only gene abundances or also the composition of the contributing NS sublineages exhibited seasonal dynamics, we followed the changes in one selected subplot over time. We chose one of the 30 available subplots (see sampling scheme in Regan et al., 2014) that exhibited the most pronounced dynamics in 16S rRNA gene abundances for NS-like NOB (**Figure 4C**). We compared shifts in the relative activity of OTUs by plotting their relative abundances against each other, setting the total abundance to 1 (**Figure 4A**). The proportions of the NS OTU abundances did not change during the first half of the year. From August on, the relative abundance of OTU03 in particular increased at each subsequent sampling date until the end of the year. While this effect was observed for the whole dataset (**Figure 4B**), it was especially pronounced in this location, suggesting spatial heterogeneity of species distribution.

## Discussion

### Temporal Dynamics and Metabolic Activity of NOB

To provide insight into the temporal dynamics of active organisms and to help identify different sublineages of dominant NS-like NOB, the abundance of 16S rRNA as a proxy for metabolic activity was assessed by an Illumina sequencing approach. Discrepancies in the direct comparison of gene abundances on a DNA level to metabolic activity at an rRNA level are attributable to the fact that gene abundances do not necessarily indicate growth or reflect activity at the RNA level (Chen et al., 2008; Offre et al., 2009; Blazewicz et al., 2013; Placella and Firestone, 2013; Daebeler et al., 2014). Marginally higher abundances of NS-assigned 16S rRNA sequences on the RNA level (Supplementary Figure S1), compared with lower *Nitrospira* rRNA 16S gene abundances on the DNA level during autumn (Supplementary Table S2) may be explained by high activity of a few organisms in cell-maintenance or in the investigated processes (Blazewicz et al., 2013). In the first half of the year, the reverse was observed. This may indicate that large numbers of NS-like NOB were inactive under suboptimal growth conditions, in a state of starvation and dormancy (Ettema and Wardle, 2002). Enzyme stability (Chen et al., 2007; Ke et al., 2013) or the constitutive expression of multiple gene copies (Poly et al., 2008; Lücker et al., 2010) could be important prerequisites for an immediate reaction to changing environmental conditions such as the sporadic availability of substrate (Blazewicz et al., 2013).

Temporal analysis demonstrated pronounced seasonal dynamics of AO and NO both with respect to their abundances and to the numerical dominance of AOA within the AOs and NS within the NOs at all measured dates (Supplementary Table S2), corresponding to previous studies (Leininger et al., 2006; Adair and Schwartz, 2008; Meyer et al., 2013; Ollivier et al., 2013; Stempfhuber et al., 2014). The higher abundance of genes involved in particular transformation processes may result not only from ammonia- or nitrite-oxidation, but also from potential mixotrophic growth, as proposed for NS and AOA (Prosser and Nicol, 2008; Jia and Conrad, 2009). The high standard deviations in gene copy numbers at one sampling date therefore highlight the importance of supplementing temporal analysis with spatial structure analysis in the field by the identification of local hotspots.

### Temporal Dynamics of Spatial Niche Differentiation Amongst NOB

Functionally complementary microbial groups often differ in their responses to environmental changes, shaping functional niches (Maixner et al., 2006). Studies have addressed spatial niche differentiation patterns of functionally redundant organisms often co-existing at the same spatial scale (Schauss et al., 2009; Schleper, 2010; Wertz et al., 2012; Ollivier et al., 2013) or differing in their spatial distribution (Krause et al., 2010, 2013). Our data showed seasonally varying patterns of niche differentiation: spatial niche separation between NS and NB was most evident at our study site in April, as large patches of high gene abundance were clearly spatially discriminated (**Figures 2A1,B1**), whereas homogeneous and congruent abundance patterns for both NS and NB were found in August, indicating co-occurrence at the same spatial scale (**Figures 2A4,B4**). We attribute these co-occurrence patterns to different adaptations to substrate concentrations, making possible the co-existence of NB and NS by reduced “interspecific” competition (Hibbing et al., 2010): it has been suggested that NB as *r*-strategists exhibit high growth rates and activity and may therefore out-compete NS under high nitrite levels (Schramm et al., 1999; Maixner et al., 2006), while NS may have a competitive advantage over NB under nitrite-limitation (Lücker et al., 2010). In November, rather undifferentiated and very patchy patterns were detected for NS and NB, without areas of clear spatial separation or congruence (**Figures 2A6,B6**).

Nitrite concentration is usually below the detection limit in natural terrestrial systems, transformed rapidly to prevent its toxic accumulation (Burns et al., 1995; Attard et al., 2010; Xia et al., 2011; Ke et al., 2013). One can infer, however, from the absence or presence of AO spatial distribution patterns at the same investigated scale, information about the nitrite content in soil, assuming that substrate availability shapes the niche differentiation patterns of NOB. Unfortunately, we could not visualize environmental variables for April and November that could explain the spatial distribution of NOB phyla. Nevertheless, we may speculate that the absence of ammonia-oxidizers at the observed spatial scale in April (**Figure 2**) suggests that nitrite formation derived from AO was low. Under such nitrite substrate-limited conditions, other niche determining factors operating at the investigated scale may have been more important. For example, the measured high soil moisture content in April (Regan et al., 2014) suggests that oxygen status could have influenced spatial niche separation. NB are presumed to prefer high oxygen conditions and thus compete with heterotrophic organisms or AO for oxygen (Kim and Kim, 2006), while NS could occupy spatial niches with extremely low oxygen content (Gieseke et al., 2003; Lücker et al., 2010). However, especially under low nitrite/nitrate conditions, NOB can switch to nitrite reduction, i.e., the reduction of nitrate to nitrite, which can be catalyzed by NXR (Sundermeyer-Klinger et al., 1984; Bock et al., 1988; Bock and Wagner, 2006). Under anoxic conditions, some NB may also perform the complete denitrification process (Freitag et al., 1987). The ability of NB to also exhibit heterotrophic growth could then provide a competitive advantage over NS (Freitag et al., 1987; Lücker et al., 2010).

### Temporal Dynamics of Spatial Niche Differentiation Amongst Sublineages of NOB

Niche differentiation has been demonstrated within genera and species of NOB. Putative shifts within NB-like NOBs, however, would not have been captured by our approach, since the V3 region of the 16S rRNA gene might not be sufficient to distinguish between the phylogenetically highly similar NB species (Freitag et al., 2005; Alawi et al., 2009), closely related to *Bradyrhizobia* (Orso et al., 1994). Thus we restricted our subsequent phylogenetic analyses to *Nitrospira* community composition for which the co-existence of up to three distinct sublineages has been reported (Freitag et al., 2005; Maixner et al., 2006; Lebedeva et al., 2008), in line with our results. NS OTU01 and OTU02 were phylogenetically placed in close proximity to cultured or enriched representatives of different sublineages (**Figure 3**, see Supplementary Table S4 for details): sublineage VI (Lebedeva et al., 2011), sublineage II (Ehrich et al., 1995; Daims et al., 2001) and sublineage I (Lücker et al., 2010). Sublineages I (Spieck et al., 2006) and II, correlated to the presence of AOA in volcanic grassland soils (Daebeler et al., 2014), are adapted to low substrate and oxygen concentrations (Maixner et al., 2006; Wertz et al., 2012; Ke et al., 2013). OTU03 of NS was affiliated to *Ca. Nitrospira bockiana* with 94% similarity (**Figure 3**), and similar substrate preferences that hold true for *Ca. Nitrospira bockiana* as cultured representative may also apply to other members of sublineage V (Lebedeva et al., 2008), such as the inability to be stimulated by organic substrates or to take up pyruvate. NS OTU03 may exhibit similar characteristics. However, transferring knowledge on habitat preferences attained from cultivated species or enrichment studies to pathways and metabolism of microorganisms in their natural habitats has to be handled with care (Regan et al., 2003; Prosser and Nicol, 2012).

We therefore addressed the question of whether or not the microbial structure at sampling sites with high gene abundances is fundamentally different from that at sites of low abundance with regard to their NS OTU composition (**Figure 4**). We selected the subplot with the most pronounced changes in NS abundance. Despite varying gene abundances, the community composition and its relative metabolic activity did not change during the first half of the year, implying the co-existence of sublineages under substrate-limitation. In the second half of the year, the relative proportion of OTU03 in particular, affiliated with sublineage V (Lebedeva et al., 2008), increased. We speculate that nitrite operates as a niche determining factor in “intraspecific” competition and may have caused shifts in the relative abundances of OTUs and affiliated sublineages from August on (Maixner et al., 2006), as even sublineages of the genus NS have been proposed to exhibit different preferences for nitrite concentrations (Grundmann and Debouzie, 2000; Maixner et al., 2006).

### Spatial Interactions of Nitrifying Organisms

Studies on nitrifiers at spatial ranges from μm (Maixner et al., 2006) to the landscape scale (Grundmann and Debouzie, 2000; Bru et al., 2011) have demonstrated that the factors influencing spatial dependency operate at different scales: soil texture or land management practices operate at larger spatial scales while, for example, vegetation, can operate at smaller scales (Ettema and Wardle, 2002; Ritz et al., 2004). Nitrification at some sampling dates may have occurred at nested scales which were not characterized. High nugget effects for AOA and AOB abundances at some dates imply the presence of unmeasured variance at smaller scales (Supplementary Table S3) (Steffens et al., 2009). The ranges of spatial dependence of the abundance data in this study (Supplementary Table S3) were, however, similar to spatial autocorrelations ranging from 1.4 to 7.6 m for AOA and AOB in a previous study in the same region (Keil et al., 2011), and corresponded also to those found in studies at mm to m scales (Nunan et al., 2003; Franklin and Mills, 2009).

**FIGURE 5:**
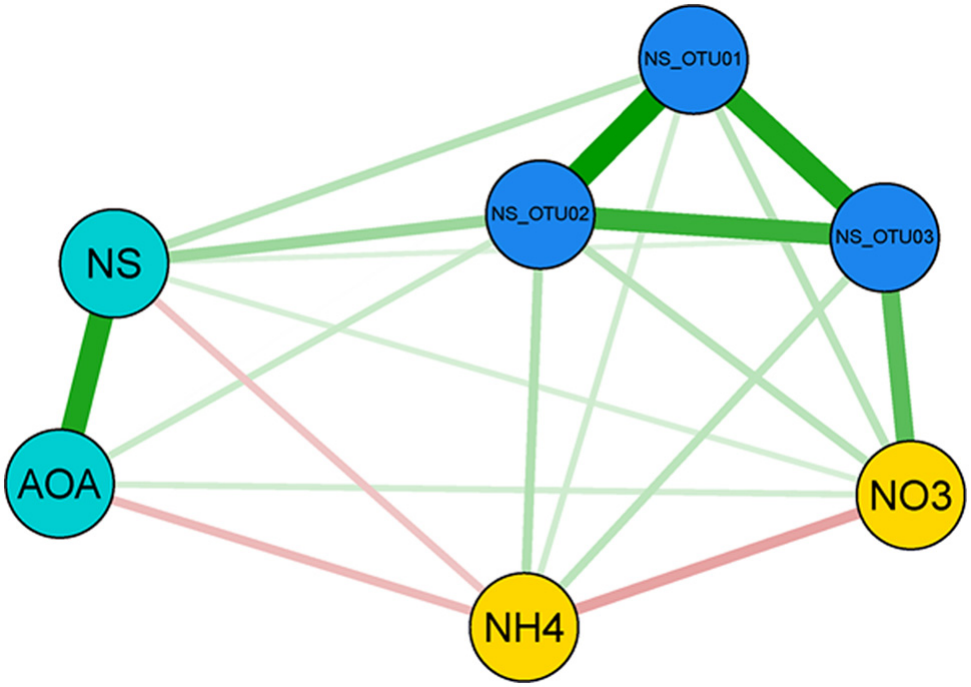
**Network analysis of interactions between NS-assigned OTUs, gene abundances and nitrification-associated nitrogen-pools in October.** Depicted are Pearson correlations between three parameter groups for sampling date October: gene abundances (light blue circles), *Nitrospira* OTUs 01-03 (dark blue circles) and nitrate and ammonium concentrations (yellow circles), respectively. Edges between the nodes are weighted according to the correlation strength. Positive coefficients are colored in green, negatives are displayed in red. AOA, Ammonia-oxidizing archaea; NS, *Nitrospira*-like nitrite-oxidizing bacteria (NOB).

**FIGURE 6:**
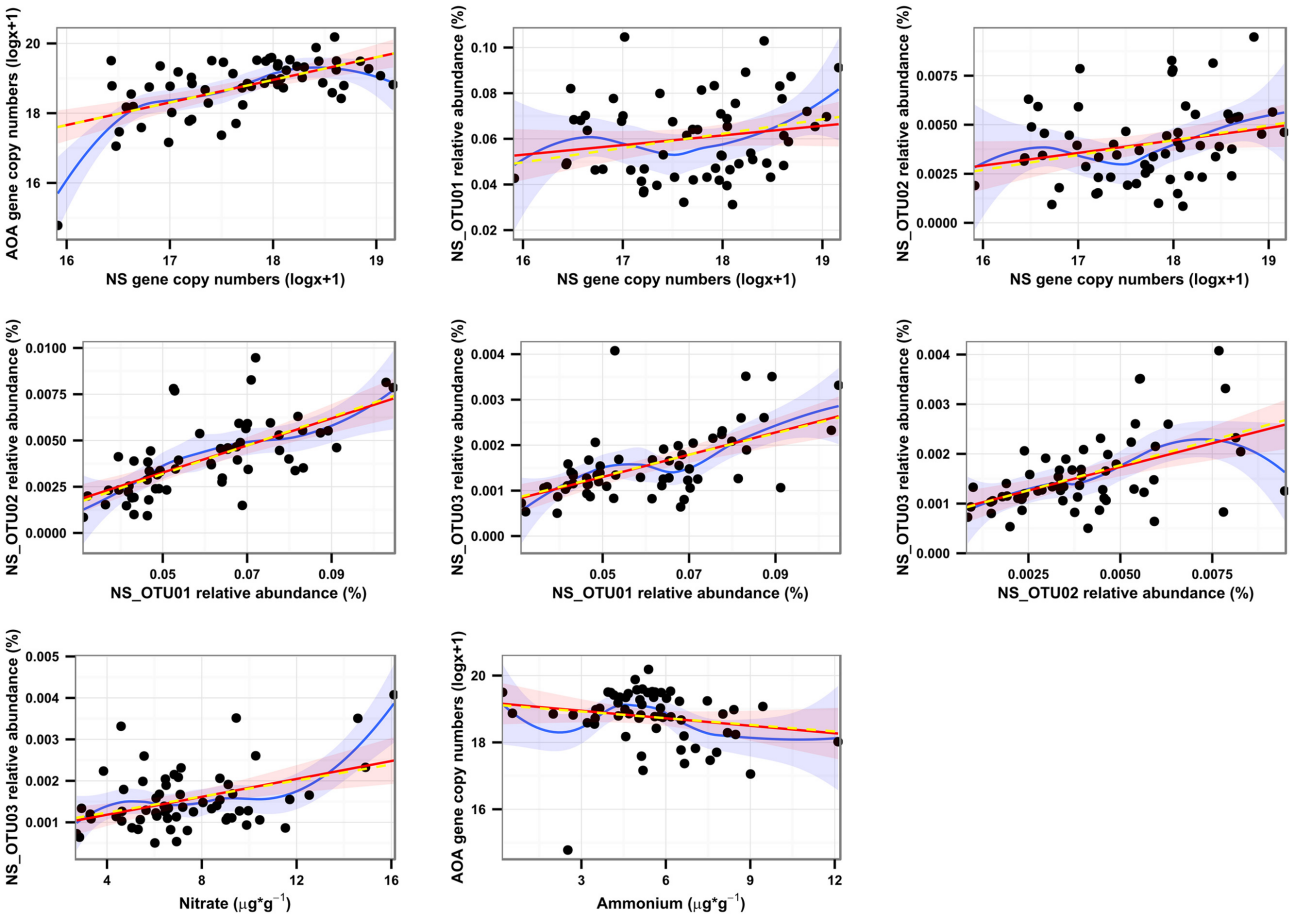
**Univariate linear models between pairs of variables in October.** Pairs of variables were selected from the network analysis (**Figure 4**) to show additional support for our conclusions after accounting for spatial autocorrelation. Red lines indicate the uncorrected, Gaussian regression models, whereas yellow, dashed lines represent the same models after correction for spatial autocorrelation. Blue lines are derived from Loess fits. All models are significant at *p* < 0.05, except for AOA/Ammonium (*p* > 0.1) and NS_OTU02/NS (*p* = 0.0565), which, however, show significant spearman rank correlations, possibly pointing at significant, non-parametric models. Although the model improvements for all variables were very small according to AIC shifts, NS and the NS OTUs 01 and 02 were best described with exponential variograms and NS OTU03 with the spherical variogram. For nitrate and AOA, no spatial model led to model improvements.

Surprisingly, our spatial analysis at the plot scale did not confirm the hypothesis that nitrification could be attributed mainly to a close functional interaction reflected by the spatial dependence of AOB and NOB, although many studies have reported their functional interaction (Mobarry et al., 1996; Schramm et al., 1999; Abeliovich, 2006; Xia et al., 2011; Wertz et al., 2012). AOB and NB have been shown to dominate nitrification under high substrate-conditions (Shen et al., 2008; Jia and Conrad, 2009; Di et al., 2010; Wertz et al., 2012; Ke et al., 2013). In contrast, the congruent spatial distributions of AOA and NS and their positively correlated abundances in autumn (*r* = 0.574 for Oct.; **Figure 2**; Supplementary Table S5), strongly suggest an interaction of AOA and NS in performing the sequential transformation steps of nitrification. This is further supported by reports on the co-occurrence of AOA and NS in the same soil compartments (Lebedeva et al., 2011; Ke et al., 2013; Daebeler et al., 2014). Since a sensitivity of AOA to nitrite accumulation was demonstrated recently for *Nitrosotalea* isolates, a close mutualistic relationship between AOA and NOB seems reasonable (Lehtovirta-Morley et al., 2014). Although the exact mechanisms are still under investigation, it has been demonstrated that both AOB and AOA are able to catalyze the transformation of ammonia to nitrite (e.g. Tourna et al., 2011). Efficiency and kinetics of ammonia-oxidation and consequently the release of nitrite might, however, vary between distinct phyla and environmental conditions (Ward, 2011). Thus it can be speculated that NOB respond to different levels of nitrite that are either determined by kinetics of ammonia-oxidation or by the relative distance of NOB to the source of their substrate (Maixner et al., 2006), according to their distinct preferences for nitrite concentrations. The temporal and spatial interaction of AOA and NS and their linkage to ammonium- and nitrate-pools were further supported by a Pearson-coefficient-based network analysis for October (**Figure 5**), when congruent spatial patterns of AOA and NS were most pronounced (**Figures 2** and **6**; Supplementary Table S5) and all investigated molecular markers were highly correlated with each other, which was observed only in October (Supplementary Figure S3). Several significant, positive pairwise correlations were detected in October. Correlations between nitrate and NS OTU03, AOA and NS, as well as NS and NS OTUs 01 and 02, respectively, were all found to be significant at *p*_adjusted_ < 0.05, and remained significant after correction for spatial autocorrelation. Furthermore, strongly positive correlations of AOA and NB were observed as well (April: *r* = 0.576, October: *r* = 0.561), but their interaction at the spatial scale could not be identified by our geostatistical analyses (Supplementary Table S5).

Nitrate concentration was positively connected most clearly with OTU03 in October (*r* = 0.42; Supplementary Figure S2), which hints at the active participation of sublineage V (**Figure 3**) in the production of nitrate and for subsequent nitrite oxidation from August on (**Figure 4**). The ability of most NOB to simultaneously convert nitrate to nitrite implies that their performance can influence the nitrate pool in different directions, impeding determination of clear positive or negative correlations (Supplementary Figure S2). The positive correlation of AOA and nitrate (**Figure 5**) was likely due to the direct connection of AO and NO processes, the former delivering the product for the latter transformation step. AOA abundance was strongly negatively correlated to ammonium content, which corresponds to their spatial distribution patterns, which varied inversely (**Figures 2C5,E5**), indicating consumption of ammonia as substrate by AOA (Schleper and Nicol, 2010; Ke et al., 2013). The negative correlation of nitrate and ammonium (*r* = 0.233; **Figure 5**; Supplementary Figure S2) could be due to a decline in the ammonia pool by AO, resulting in an increase in nitrate content due to NO. This confirms that the complete nitrification process based on interactions between ammonia- and nitrite-oxidizers can be followed at the investigated scale only at very limited periods during the year. It must be considered, however, that nitrification at other dates may be performed by organisms that catalyze complete nitrification (commamox) that have not been assessed by our study of spatial interaction patterns (Daims et al., 2015; van Kessel et al., 2015).

Different growth strategies such as potential mixotrophy or heterotrophy may obscure the interactions between AOA and NS. Consequently, the utilization of alternative substrates (Prosser and Nicol, 2008, 2012; Tourna et al., 2011) for energy production and assimilation of different carbon sources (Lehtovirta-Morley et al., 2013) must also be taken into account. The potential for mixotrophic growth (Rogers and Casciotti, 2010; Lehtovirta-Morley et al., 2014) could increase the competitiveness of AOA and NS over their counterparts by providing a growth advantage and assuring their greater flexibility in reacting to suboptimal substrate-limited conditions. An increase of organic material, as observed in autumn due to plant litter, may further support the growth of mixotrophic organisms (Brown et al., 2013). Differences in preferences for, e.g., organic compounds or other characteristics have been reported even within particular AOA species in soils (Offre et al., 2009; Hatzenpichler, 2012; Lehtovirta-Morley et al., 2014) and for ecotypes of *Nitrospira* (Maixner et al., 2006). This heterogeneity could affect patterns of spatial distribution and inhibit correlation of abundances to environmental parameters. Given this, it becomes necessary to identify drivers which may influence nitrifiers directly or indirectly via changing substrate availability or ammonia sources (Prosser and Nicol, 2012). AOA, for example, prefer mineralized nitrogen, derived from decaying plant material, which is the main source of inorganic nitrogen at the end and before the start of the vegetation period, rather than ammonium directly applied by fertilization (Offre et al., 2009; Levičnik-Höfferle et al., 2012).

Even occasional mowing or grazing may influence nitrogen availability and consequently the microbes performing nitrification (Patra et al., 2005, 2006). We assumed, therefore, that the a mowing event in August (2 weeks before sampling) affected the observed nitrification activity in autumn (Both et al., 1992), uncoupling the plants’ competition for substrate, thereby enabling AO to better access the ammonium pools in soil (Wolters et al., 2000; Hamilton and Frank, 2001; Patra et al., 2006; Le Roux et al., 2008; Kuzyakov and Xu, 2013). The heterogeneous ammonium distribution may also be linked to plant diversity, as a strong spatial distribution pattern of legumes was observed mainly in October at the site (Regan et al., 2014).

This study presents evidence for both temporal and spatial correlation of AOA and *Nitrospira* in an unfertilized grassland site, indicating their interrelationship in performing the nitrification process over one growing season. The obtained results, however, are based on a 1-year study. Thus, it would be important to assess spatial interaction patterns at larger temporal scales to confirm stability of the observed patterns. However, *Nitrobacter* and ammonia-oxidizers might interact at scales not covered by our study, below the m^2^ range, and may require subsequent studies using microscopic techniques.

We demonstrated an interaction of AOA and NS under unfertilized conditions, and it would be interesting to extend this approach to sites under high land-use intensity with different fertilization practices to compare both the major actors and their interactions (Keil et al., 2011). Recently, alternative possibilities have been described for nitrifiers to gain ammonia using cyanate as substrate (Stein, 2015). It has been demonstrated that ammonium derived from cyanate transformation by NS can be used by ammonia-oxidizing microbes (Palatinszky et al., 2015); such alternative feedback processes may exist between functional guilds of nitrification and play an important role for the stabilization of nitrifier networks mainly in fertilized soils.

## Author Contributions

BS and TR-H conducted and interpreted the experiments and wrote the manuscript. KR performed the soil sampling and sample preparation as well as critical revision of the draft. AK contributed to geostatistical analyses and data interpretation. PK and JS contributed to phylogenetic and statistical analyses. SM performed soil sampling and was responsible for the conception of the experiment. JO, MF, EK, and MS were involved in the conception of the experiments and final approval of the manuscript.

## Conflict of Interest Statement

The authors declare that the research was conducted in the absence of any commercial or financial relationships that could be construed as a potential conflict of interest.

## Footnotes

1 http://www.biodiversity-exploratories.de/startseite/

2 http://www.R-project.org
