# Supplementary material for: Spatial Interaction of Archaeal Ammonia-Oxidizers and Nitrite-Oxidizing Bacteria in an Unfertilized Grassland Soil
Source: Front Microbiol. 2016 Jan 22;6:1567. doi: 10.3389/fmicb.2015.01567 (PMC4722141; doi:10.3389/fmicb.2015.01567)
Supplement: Supplementary file 1 [file Presentation_1.PDF]

Supplementary Material

Spatial interaction of archaeal ammonia-oxidizers and nitrite-oxidizing bacteria in an unfertilized grassland soil

Barbara Stempfhuber*, Tim Richter-Heitmann, Kathleen M. Regan, Angelika Kölbl, Pia K. Wüst, Sven Marhan, Johannes Sikorski, Jörg Overmann, Michael W. Friedrich, Ellen Kandeler, Michael Schloter

*** Correspondence:** Barbara Stempfhuber: barbara.stempfhuber@helmholtz-muenchen.de

1 Supplementary Experimental Procedures

Study site description and sampling design

In the frame of the Scalemic project, the following abiotic soil parameters were determined and did not change over the season: pH (6.7), carbon (66.0 mg g^{-1}) and nitrogen (7.0 mg g^{-1}) content, bulk density and soil texture. Extractable organic carbon was in the range between 208.25 µg g^{-1} and 100.19 µg g^{-1}, decreasing over the year. Extractable organic nitrogen ranged from 1.69 µg g^{-1} to 11.04 µg g^{-1} with lowest values in August and November. Highest values for NH_4^+ and NO_3^- have been detected in April (15.70 µg g^{-1} and 17.99 µg g^{-1}, respectively), lowest values in October (5.43 µg g^{-1} and 7.40 µg g^{-1}, respectively). Soil moisture was dynamic over the year, exhibiting highest water content in April (57.56%) and lowest soil moisture contents in May and October (27.97% and 26.79%). For further information see Regan et al. (2014) and the corrigendum Regan et al. (2015).

At each sampling date, before soil cores were collected, 20 cm x 20 cm grids were centered over each of the 60 sampling points. Aboveground biomass was removed from each grid by cutting all visible plants at ground level. Samples were then sorted into the following categories: litter (dead leaves and other dead plant matter on soil surface), grasses (*Poaceae*), legumes, forbs, bryophytes, and *Rhinanthus minor*. Vegetation coverage for all sampling dates for the three most abundant plant categories (grasses, forbs, legumes) and litter mass was calculated as g per 400 cm^2 grid. Grasses dominated at the first three sampling dates, but were rather low in abundance from August to November. Forbs followed the pattern of grasses, but with lower abundance than grasses in May and June. Legumes were the only plant group to increase after mowing, appeared from June on and were highest in October. Litter mass exhibited a different pattern; it declined from April to June, increased after mowing and continued to increase at each of the last three sampling dates. Details can be found in Regan et al. (2014).

Geostatistical analysis

Geostatistical semivariogram analyses were carried out using the g-stat package (Pebesma, 2004) for the R environment (R 3.0.2, RDevelopmentCoreTeam, 2008). Nugget (intercept at the origin), sill (the plateau at which the distance based variance values levels off, representing the maximum semivariance) and range (maximal distance of spatial autocorrelation) have been determined by semivariogram analyses and used for calculation of measures of spatial dependence and variance. P-sill is defined as the part of total variance that is spatially explained. The degree of spatial dependence is calculated by dividing nugget by the sum of nugget and sill (Fortin and Dale, 2005; Steffens et al., 2011). Values below 25% resulting from low nugget values related to maximal semivariance, indicate the presence of spatial dependency, whereas high values above 75% would point to the absence of spatial dependency (Cambardella et al., 1994; Steffens et al., 2009; Steffens et al., 2011). Our results (values below 49%) indicated a spatial dependence for all measured variables at sampling dates when a model could be fitted (Steffens et al., 2009). An appropriate model was fitted to each experimental semivariogram using the gstat fitting routine. Spherical models were tested first, as they would best explain spatial dependencies (Berner et al., 2011). Furthermore, exponential models were tested if no spherical model could be fitted. The fitting of a geostatistical model indicated that the selected spatial scale of the study might be appropriate to describe the spatial distribution of the observed parameters (Steffens et al., 2009). Step widths between 0.6 and 0.8 m were applied.

The display of spatial distribution of AOA and AOB variables reflects the rather weak spatial dependence after fitting of different settings to construct kriged maps. Nevertheless, the maps display a rough distribution of variables, for visual comparison of e.g. co-localization and hence are essential for gaining deeper insights in the interaction of functional groups under observation.

Berner, D., Marhan, S., Keil, D., Poll, C., Schützenmeister, A., Piepho, H.-P., and Kandeler, E. (2011). Land-use intensity modifies spatial distribution and function of soil microorganisms in grasslands. Pedobiologia 54, 341-351. doi: http://dx.doi.org/10.1016/j.pedobi.2011.08.001.

Cambardella, C.A., Moorman, T.B., Parkin, T.B., Karlen, D.L., Novak, J.M., Turco, R.F., and Konopka, A.E. (1994). Field-Scale Variability of Soil Properties in Central Iowa Soils. Soil Sci. Soc. Am. J. 58, 1501-1511. doi: 10.2136/sssaj1994.03615995005800050033x.

Fortin, M.-J., and Dale, M.R.T. (2005). Spatial analysis: a guide for ecologists. Cambridge University Press.

Pebesma, E.J. (2004). Multivariable geostatistics in S: the gstat package. Computers & Geosciences 30, 683-691.

Regan, K.M., Nunan, N., Boeddinghaus, R.S., Baumgartner, V., Berner, D., Boch, S., Oelmann, Y., Overmann, J., Prati, D., and Schloter, M. (2015). Corrigendum to “Seasonal controls on grassland microbial biogeography: are they governed by plants, abiotic properties or both?”[Soil Biology and Biochemistry 71 (April 2014), 21–30]. Soil Biology and Biochemistry 86, 212-214.

Regan, K.M., Nunan, N., Boeddinghaus, R.S., Baumgartner, V., Berner, D., Boch, S., Oelmann, Y., Overmann, J., Prati, D., Schloter, M., Schmitt, B., Sorkau, E., Steffens, M., Kandeler, E., and

Marhan, S. (2014). Seasonal controls on grassland microbial biogeography: Are they governed by plants, abiotic properties or both? Soil Biology and Biochemistry 71, 21-30. doi: http://dx.doi.org/10.1016/j.soilbio.2013.12.024.

Steffens, M., Kölbl, A., Giese, M., Hoffmann, C., Totsche, K.U., Breuer, L., and Kögel-Knabner, I. (2009). Spatial variability of topsoils and vegetation in a grazed steppe ecosystem in Inner Mongolia (PR China). Journal of Plant Nutrition and Soil Science 172, 78-90. doi: 10.1002/jpln.200700309.

Steffens, M., Kölbl, A., Schörk, E., Gschrey, B., and Kögel-Knabner, I. (2011). Distribution of soil organic matter between fractions and aggregate size classes in grazed semiarid steppe soil profiles. Plant and Soil 338, 63-81. doi: 10.1007/s11104-010-0594-9.

2 Supplementary Figures and Tables

2.1 Supplementary Figures

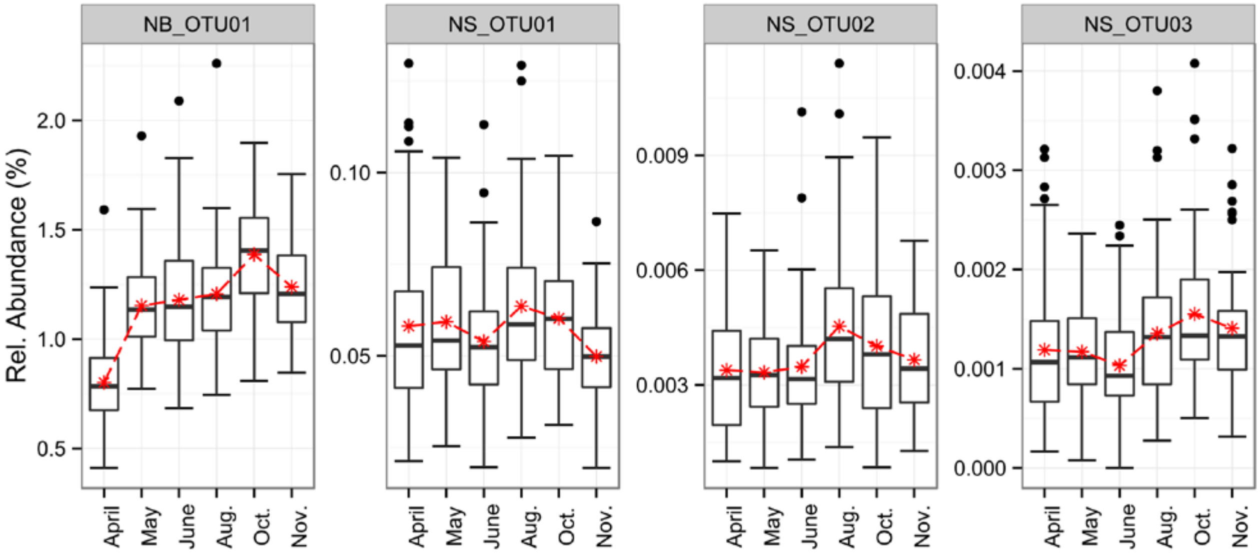

Supplementary Figure S1. Boxplots for seasonal dynamics of nitrite-oxidizing bacteria associated OTUs, showing relative read abundances.

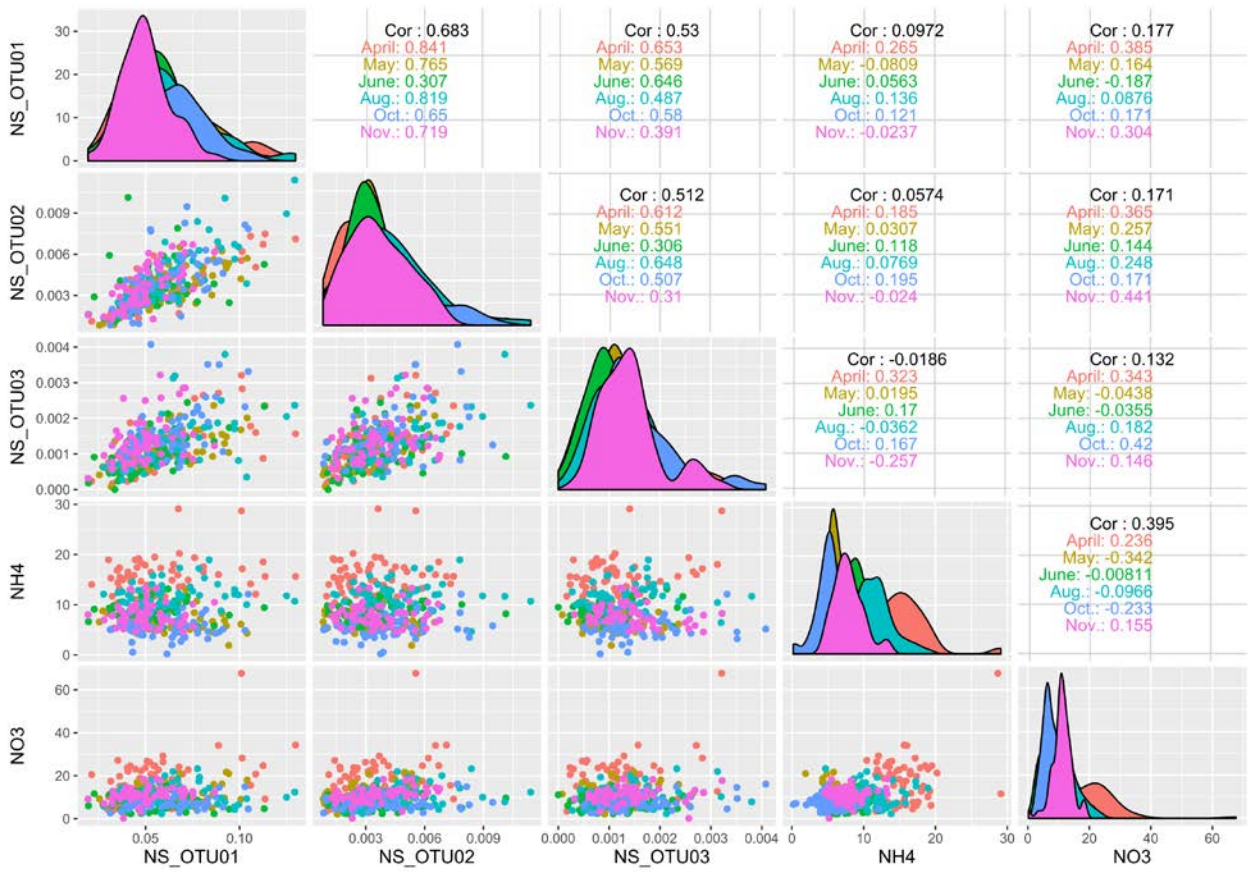

Supplementary Figure S2. The diagram shows pairwise comparisons of NS-like-nitrite oxidizing bacteria-associated OTU-abundances as well as nitrate and ammonium concentrations in the soil. Each row/column represents one of the 5 parameters, with the diagonal showing density plots. The lower triangle of the plot matrix consists of scatterplots, with the corresponding Pearson correlation coefficients appearing in the upper triangle. Data is always colored according to the sample dates. The scale for the OTU is representing percentage abundances; for ammonium and nitrate, concentrations (given in µg N per g soil dry weight) are used.

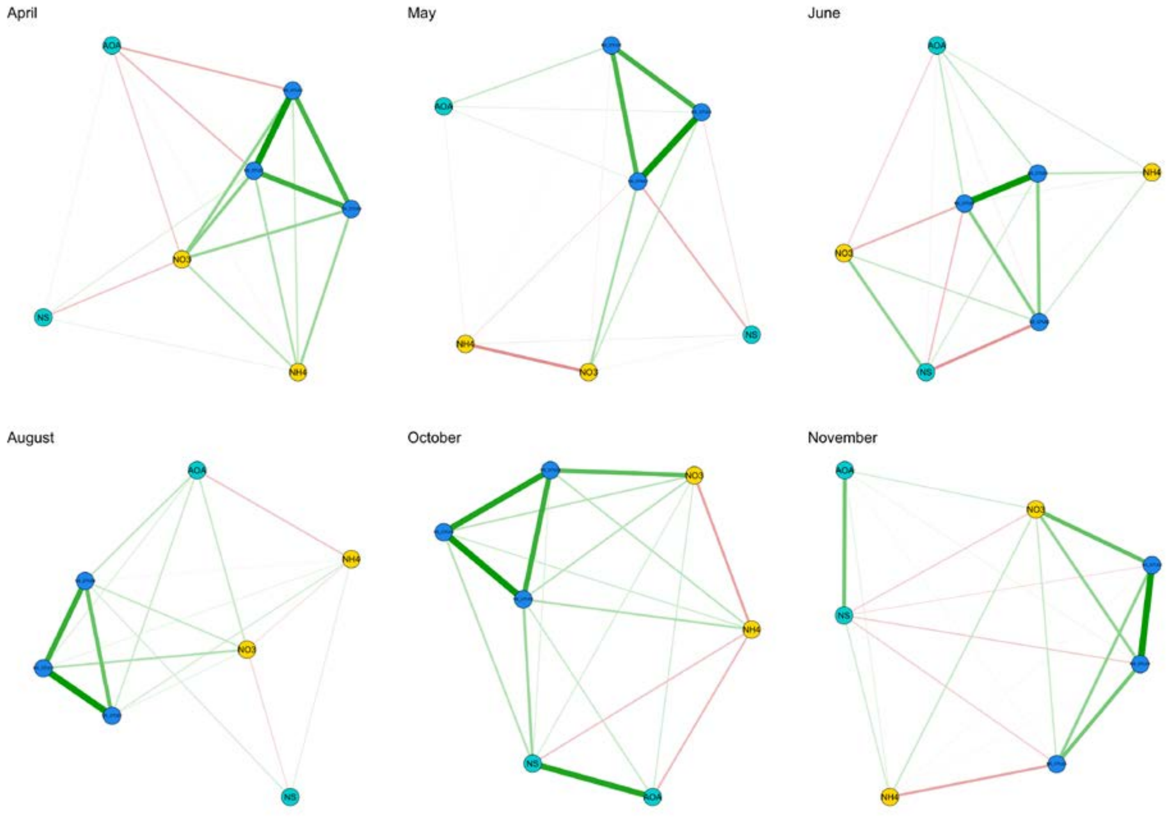

Figure S3: **Network analysis of interactions between NS-assigned OTUs, gene abundances and nitrification-associated nitrogen-pools**.

Depicted are Pearson correlations between three parameter groups for all sampling dates: gene abundances (light blue circles), *Nitrospira* OTUs 01-03 (dark blue circles) and nitrate and ammonium concentrations (yellow circles), respectively. Edges between the nodes are weighted according to the correlation strength. Positive coefficients are colored in green, negatives are displayed in red. AOA = Ammonia-oxidizing archaea, NS = *Nitrospira*-like nitrite-oxidizing bacteria (NOB).

2.2 Supplementary Tables

Supplementary Table S1. Thermal profiles, primer and standards used for real-time PCR quantification of the following genes: *amoA* (AOA), *amoA* (AOB), *nxrA* (NB) and 16S rRNA genes (NS). AOA = ammonia-oxidizing archaea, AOB = ammonia-oxidizing bacteria, NB = *Nitrobacter*-like, NS = *Nitrospira*-like.

Target gene	Standard source	Primer	Primer reference	Thermal profile	No. of cycles
amoA (AOA)	Fosmid clone 54d9	amo19F	Leininger et al., 2006	94°C/45 s, 55°C/45 s, 72°C/45 s	40
		CrenamoA16r48x	Schauss et al., 2009		
amoA (AOB)	*Nitrosomonas sp.*	amoA1F	Rotthauwe et al., 1997	94°C/60 s, 58°C/60 s, 72°C/60 s	40
		amoA2R	Rotthauwe et al., 1997		
nxrA (NB)	*Nitrobacter hamburgensis*	F1norA	Poly et al., 2008	94°C/30 s, 55°C/30 s, 72°C/30 s	40
	X14 (DSMZ 10229)	R2norA	Wertz et al., 2008		
16S rRNA gene (NS)	*Nitrospira* 16S rRNA gene	Nspra 675f	Graham et al., 2007	94°C/30 s, 64°C/30 s, 72°C/60 s	40
	Accession No. FJ529918	Nspra 746r	Graham et al., 2007		

Supplementary Table S2. Gene abundances for *amoA* (AOA), *amoA* (AOB), *nxrA* (NB) and 16S rRNA genes (NS) in copy numbers per g of soil (dry weight) at different sampling dates. Values represent mean values for the complete plot at the respective date including corresponding standard deviations. AOA = ammonia-oxidizing archaea, AOB = ammonia-oxidizing bacteria, NB = *Nitrobacter*-like, NS = *Nitrospira*-like. Asterisks indicate transitions between months which are statistically significant based on two models (i) linear Gaussian mixed models with time as random effect and corrected for spatial autocorrelation, but not for heteroscedasticity; and (ii) generalized linear models under the negative binomial distribution with correction for heteroscedasticity, but not for temporal and spatial random effects. Significance levels: * < 0.05, ** < 0.01, *** < 0.001, corrected for multiple testing.

**Gene abundances** (copies g^{-1} soil dw)	**Date**					
	April	May	June	August	October	November
nxrA (NOB-NB)	$8.07\ 10^5 \pm 2.10\ 10^6$	$1.97\ 10^7 \pm 8.90\ 10^7$ **/*	$6.04\ 10^6 \pm 3.19\ 10^7$	$5.25\ 10^5 \pm 4.80\ 10^5$ **/***	$4.53\ 10^5 \pm 7.38\ 10^5$	$9.12\ 10^5 \pm 8.40\ 10^5$ */***
16S rRNA gene (NOB-NS)	$5.19\ 10^7 \pm 3.38\ 10^7$	$1.01\ 10^8 \pm 9.71\ 10^7$ -/**	$9.46\ 10^7 \pm 5.88\ 10^7$	$6.79\ 10^7 \pm 4.99\ 10^7$	$6.37\ 10^7 \pm 4.57\ 10^7$	$1.05\ 10^8 \pm 5.99\ 10^7$ -/**
amoA (AOA)	$3.08\ 10^8 \pm 1.69\ 10^8$	$3.78\ 10^8 \pm 3.18\ 10^8$	$3.24\ 10^8 \pm 3.08\ 10^8$	$1.15\ 10^8 \pm 1.11\ 10^8$ ***/**	$1.81\ 10^8 \pm 1.08\ 10^8$	$5.34\ 10^8 \pm 4.26\ 10^8$ ***/***
amoA (AOB)	$2.58\ 10^6 \pm 3.54\ 10^6$	$1.68\ 10^6 \pm 2.84\ 10^6$	$1.02\ 10^6 \pm 8.19\ 10^5$	$3.46\ 10^6 \pm 3.25\ 10^6$ ***/***	$3.30\ 10^6 \pm 4.33\ 10^6$	$1.27\ 10^6 \pm 6.41\ 10^5$

Supplementary Table S3. Variogram parameters of gene abundances for *amoA* (AOA), *amoA* (AOB), *nxrA* (NB) and 16S rRNA genes (NS) at different sampling dates. Nugget, sill and range values are derived from fitted spherical models; step widths between 0.6 and 0.8 m were applied. Data sets to which no model could be fit ted, are indicated with “-“. AOA = ammonia-oxidizing archaea, AOB = ammonia-oxidizing bacteria, NB = *Nitrobacter*-like, NS = *Nitrospira*-like.

Gene	Variogram details	Date					
		April	May	June	August	October	November
nxrA (NOB-NB)	Nugget	1.60	-	-	100.36	-	0.12
	p-Sill	2.96	-	-	261.59	-	0.95
	Sill	4.56	-	-	361.95	-	1.07
	Range [m]	4.5	-	-	7.7	-	12.3
	Nugget / (Nugget + Sill)[a]	26.0	-	-	21.7	-	10.1
	p-Sill / Sill[b]	64.9	-	-	72.3	-	88.8
16S rRNA gene (NOB-NS)	Nugget	171.19	3700.50	2966.92	217.77	351.60	208.79
	p-Sill	1233.20	7522.09	433.67	88.89	4930.48	103.14
	Sill	1404.39	11222.59	3400.59	306.66	5282.08	311.93
	Range [m]	4.6	8.1	7.7	2.9	21.2	1.2
	Nugget / (Nugget + Sill)[a]	10.9	24.8	46.6	41.5	6.2	40.1
	p-Sill / Sill[b]	87.8	67.0	12.8	29.0	93.3	33.1
amoA (AOA)	Nugget	-	-	-	8997.87	8439.89	-
	p-Sill	-	-	-	5171.11	3292.38	-
	Sill	-	-	-	14168.98	11732.27	-
	Range [m]	-	-	-	12.9	4.9	-
	Nugget / (Nugget + Sill)[a]	-	-	-	38.8	41.8	-
	p-Sill / Sill[b]	-	-	-	36.5	28.1	-
amoA (AOB)	Nugget	-	280.39	0.39	-	-	0.40
	p-Sill	-	36.93	0.22	-	-	0.01
	Sill	-	317.32	0.61	-	-	0.41
	Range [m]	-	9.1	7.2	-	-	2.3
	Nugget / (Nugget + Sill)[a]	-	46.9	39.0	-	-	49.4
	p-Sill / Sill[b]	-	11.6	36.1	-	-	2.4

[a] Nugget / (Nugget + Sill) [%] = indicator for spatial distribution

[b] p-Sill / Sill [%] = degree of spatial dependence

Supplementary Table S4. *Nitrospira*-like NOB sublineages. All known *Nitrospira*-like sublineages are listed with cultured / enriched representatives and the respective publication citation. OTUs detected in this study that could be affiliated to different sublineages according to their phylogeny are listed in the last row.

Sublineage	Representative species	Publication	affiliated OTU (this study)
Sublineage I	Ca. *Nitrospira defluvii*	Spieck et al., 2006	OTU01 / OTU02
Sublineage II	*Nitrospira moscoviensis*	Ehrich et al., 1995	OTU01 / OTU02
Sublineage III	16S rRNA clones	Holmes et al., 2001	
Sublineage IV	*Nitrospira marina*	Watson et al., 1986	
Sublineage V	Ca. *Nitrospira bockiana*	Lebedeva et al., 2008	OTU03
Sublineage VI	*Nitrospira calida*	Lebedeva et al., 2011	OTU01 / OTU02

Supplementary Table S5. Correlation matrix for gene abundance data. Pearson correlation coefficients are given for each sampling date and across the complete season to display putative linear correlations of nitrifier abundances. AOA = ammonia-oxidizing archaea, AOB = ammonia-oxidizing bacteria, NB = *Nitrobacter*-like, NS = *Nitrospira*-like.

**Date**		AOA	AOB	NB	NS
April	AOA		0.255	0.576	0.055
	AOB	0.255		0.506	0.104
	NB	0.576	0.506		0.116
	NS	0.055	0.104	0.116	
May	AOA		-0.432	-0.008	-0.009
	AOB	-0.432		0.233	-0.147
	NB	-0.008	0.233		-0.089
	NS	-0.009	-0.147	-0.089	
June	AOA		0.433	0.159	-0.010
	AOB	0.433		0.265	-0.037
	NB	0.159	0.265		-0.158
	NS	-0.010	-0.037	-0.158	
August	AOA		0.523	0.473	-0.021
	AOB	0.523		0.176	-0.111
	NB	0.473	0.176		0.278
	NS	-0.021	-0.111	0.278	
October	AOA		0.239	0.561	0.574
	AOB	0.239		0.203	0.160
	NB	0.561	0.203		0.579
	NS	0.574	0.160	0.579	
November	AOA		-0.309	0.136	0.398
	AOB	-0.309		0.301	-0.112
	NB	0.136	0.301		0.123
	NS	0.398	-0.112	0.123	
all	AOA		0.092	0.290	0.196
	AOB	0.092		0.134	-0.084
	NB	0.290	0.134		0.153
	NS	0.196	-0.084	0.153	
